# Rational Design of Antirheumatic Prodrugs Specific for Sites of Inflammation

**DOI:** 10.1002/art.39232

**Published:** 2015-09-23

**Authors:** Shimobi C. Onuoha, Mathieu Ferrari, Daniele Sblattero, Costantino Pitzalis

**Affiliations:** ^1^ William Harvey Research Institute, Barts and The London School of Medicine and Dentistry, and Queen Mary University of London LondonUK; ^2^ University of Trieste TriesteItaly

## Abstract

**Objective:**

Biologic drugs, such as the anti–tumor necrosis factor (anti‐TNF) antibody adalimumab, have represented a breakthrough in the treatment of rheumatoid arthritis. Yet, concerns remain over their lack of efficacy in a sizable proportion of patients and their potential for systemic side effects such as infection. Improved biologic prodrugs specifically targeted to the site of inflammation have the potential to alleviate current concerns surrounding biologic anticytokine therapies. The purpose of this study was to design, construct, and evaluate in vitro and ex vivo the targeting and antiinflammatory capacity of activatable bispecific antibodies.

**Methods:**

Activatable dual variable domain (aDVD) antibodies were designed and constructed to target intercellular adhesion molecule 1 (ICAM‐1), which is up‐regulated at sites of inflammation, and anti‐TNF antibodies (adalimumab and infliximab). These bispecific molecules included an external arm that targets ICAM‐1 and an internal arm that comprises the therapeutic domain of an anti‐TNF antibody. Both arms were linked to matrix metalloproteinase (MMP)–cleavable linkers. The constructs were tested for their ability to bind and neutralize both in vitro and ex vivo targets.

**Results:**

Intact aDVD constructs demonstrated significantly reduced binding and anti‐TNF activity in the prodrug formulation as compared to the parent antibodies. Human synovial fluid and physiologic concentrations of MMP enzyme were capable of cleaving the external domain of the antibody, revealing a fully active molecule. Activated antibodies retained the same binding and anti‐TNF inhibitory capacities as the parent molecules.

**Conclusion:**

The design of a biologic prodrug with enhanced specificity for sites of inflammation (synovium) and reduced specificity for off‐target TNF is described. This construct has the potential to form a platform technology that is capable of enhancing the therapeutic index of drugs for the treatment of RA and other inflammatory diseases.

Rheumatoid arthritis (RA) is a systemic inflammatory condition that primarily affects synovial joints. It is characterized by persistent synovitis and destruction of bone and cartilage. RA affects ∼1% of the adult population, with a higher prevalence in the population over 60 years of age (2%) and a 3‐fold higher incidence in women 1. While the cause of the disease remains incompletely understood, it is known that proinflammatory cytokines play a role in its pathogenesis by sustaining inflammation, which leads to joint destruction 2. Key cytokines in the development of RA include tumor necrosis factor (TNF), interleukin‐1β (IL‐1β), and IL‐6. These cytokines can stimulate the production of matrix metalloproteinase (MMP) enzymes, destroying the extracellular matrix and leading to cartilage and bone damage 3. Collagenases MMP‐1 and MMP‐13 play a significant role in RA, as they are shown to be the rate‐limiting step in the process of collagen degradation 4.

In recent years, the availability of biologic drugs has revolutionized the field of RA treatment. Nonetheless, the disease continues to be linked to severe pain, depression, and impaired function, with 20–40% of patients failing to respond to current therapy 5, 6. The cost of treating RA with biologic agents is far higher than the cost of “conventional” disease‐modifying antirheumatic drugs (DMARDs) and continues to be linked to negative consequences of organ toxicity 7. Targeting TNF with monoclonal antibodies such as adalimumab (Humira; AbbVie) and infliximab (Remicade; Janssen Biologics), either alone or in combination with other DMARDs, has become the gold standard for RA therapy 8. While TNF has a highly deleterious effect in inflammatory joint diseases, it plays a vital role in the body's defenses against infection 9. In the immune response to *Mycobacterium tuberculosis*, TNF plays a critical protective role, leading to macrophage activation, cell recruitment, granuloma formation, and maintenance of granuloma integrity 10, 11, 12. Thus, systemic blockade of TNF increases the risk of tuberculosis infection and reactivation in patients with latent disease, as compared to alternative DMARD therapy 13. Although the exact mechanism behind the high number of nonresponders to anti‐TNF biologic therapy is not clear 14, it is plausible to hypothesize that a lack of efficacy may be due to suboptimal TNF blockade at sites of inflammation, which cannot be improved by increased systemic administration because of potential general toxicity when taken at doses higher than those recommended 15, 16.

A possible solution would be to develop new agents with dual specificity, in which one domain targets molecules expressed at high levels (e.g., cell adhesion molecules) at the site of disease (the synovium in the case of RA) and the other domain targets the cytokine of interest (e.g., TNF). Bispecific agents, such as dual variable‐domain immunoglobulins (DVD‐Ig) 17, could theoretically deliver higher local concentrations with lower systemic exposure. In this format, the variable domains of 2 distinct monoclonal antibodies are linked, creating a tetravalent, dual‐targeting single agent. While it has been shown that viable DVD‐Ig molecules can be identified through optimization of an antibody pair, antibody variable domain orientation, and linkers, an ongoing limitation of the technology is the lower binding affinity observed by the “inner domain” as compared to the “outer domain.” Several bodies of work have investigated the possibility of increasing the viability of the inner domain by using variable linkers, and it has been suggested that each antibody needs to be optimized individually in terms of inner/outer domain arrangement and linker length construction to derive the best molecule 18, 19.

By reversing the concept, the intrinsic reduced activity of the inner domain represents an advantage in the design of an antibody‐based “prodrug”‐targeting molecule, with an outer domain capable of targeting the inflamed synovium and an inner domain that binds TNF. We hypothesized that by varying the linker length, it would be possible to reduce the binding affinity of the inner domain to TNF in its circulating unbound form while maintaining the specificity of the outer domain for the target of interest. This, coupled with further engineering of the linker to contain an MMP‐cleavable sequence, would allow a fully functional antibody to be released and to act locally at the site of inflammation. Such a molecule, which we identify as an activatable DVD (aDVD), would have the benefits of reducing systemic toxicity while increasing the therapeutic dosage available at sites of disease, thus improving its therapeutic index.

## MATERIALS AND METHODS

### Cloning and expression of DVD antibodies

The sequences of the variable regions of anti‐human ICAM‐1 antibody and human TNF have been previously described 20, 21. Sequence data management was performed using Serial Cloner 2.6. Variable sequences were generated by gene synthesis (GenScript) and combined into various constructs using overlapping‐extension polymerase chain reaction (PCR) 22. The PCR products were cloned into the AbVec‐hIgG1 and AbVec‐hIgK vectors 23 using the restriction sites *Age* I–*Sal* I and *Age* I–*Bsi* WI, respectively. Clones were sequence‐verified prior to protein expression.

Twenty‐four hours before transfection, vectors encoding the heavy and light chains of the DVD antibody were transfected into HEK 293T cells in Dulbecco's modified Eagle's medium (DMEM) containing 10% fetal bovine serum (FBS), 100 units/ml of penicillin, 100 μg/ml of streptomycin, and 0.5 mg/ml of Geneticin. Transfection was performed with JetPrime reagent (Polyplus) according to the manufacturer's protocol. The antibodies were purified from the supernatant via affinity chromatography using protein A–Sepharose CL‐4B (GE Healthcare). DVD antibodies were biotinylated using an EZ‐Link Sulfo‐NHS‐SS biotinylation kit (Thermo‐Fisher Scientific) according to the manufacturer's protocol.

### MMP enzymatic digestion

Antibodies were incubated at 37°C at a concentration of 100 μg/ml with 35 units of recombinant MMP‐1 enzyme (Enzo Life Sciences) in 50 m*M* Tris, 0.15*M* NaCl, 10 m*M* CaCl_2_, 50 m*M* ZnCl_2_, and 0.02% Brij35. Antibodies used for kinetic analysis were digested for 1 hour at 37°C. Digestion with RA synovial fluid (SF) and RA serum was performed by incubating 500 ng of biotinylated antibody in 200 μl of fluid at 37°C for 24–72 hours in the presence of 20 μ*M* GM6001 (MMP inhibitor).

### Protein characterization

Protein purity and molecular weight were assessed by resolution in sodium dodecyl sulfate–polyacrylamide gel electrophoresis (SDS‐PAGE) reducing gels using Mini‐Protean 4–20% TGX gels (Bio‐Rad) followed by Sypro Ruby protein gel stain according to the manufacturer's instructions. Western blot analysis of antibodies digested with RA SF and serum was performed via nitrocellulose transfer. Biotinylated antibody heavy and light chains were detected using streptavidin–horseradish peroxidase (HRP).

### Quantification of anti‐TNF activity

Enzyme‐linked immunosorbent assay (ELISA) of anti‐TNF activity was performed in 96‐well plates (Thermo‐Fisher Scientific) that had been coated overnight at 4°C with 100 ng/ml of TNF in phosphate buffered saline (PBS). Plates were blocked for 2 hours at room temperature with PBS/2% bovine serum albumin and then incubated with serial dilutions of DVD antibody. Bound antibodies were detected with HRP‐conjugated anti‐human IgG antibody (Jackson Immunotools). Plates were then incubated with tetramethylbenzidine substrate (GE Healthcare), and reactions were stopped with 1*N* H_2_SO_4_. Optical absorption was measured at 450 nm. The 20% effective concentration (EC_20_) was calculated using a dose‐response nonlinear‐fit curve in GraphPad Prism v5.

Inhibition of TNF‐induced cytotoxicity was conducted in the L929 cell line. Briefly, 3 × 10^4^ cells were seeded in 96‐well plates for 18 hours at 37°C in 100 μl of DMEM supplemented with 10% FBS, 100 units/ml of penicillin, 100 μg/ml of streptomycin, and a 10 μ*M* concentration of the MMP inhibitor GM6001. The medium was then replaced with 100 μl of complete medium with 1 μg/ml of actinomycin D and 0.45 ng/ml of either TNF (Sigma) or TNF plus the antibody of interest (1:2 serial dilutions), and this was incubated for 24 hours at 37°C. We added 500 μg/ml of thiazolyl blue tetrazolium bromide in PBS (Sigma) to the wells and incubated them for 3 hours at 37°C. Medium was then removed, and the cells were resuspended in 100 μl of 90% isopropanol 10% DMSO for 15 minutes. Optical absorption was measured at 595 nm. The percentage of viable cells was calculated as follows: (OD_595 nm_ × 100)/OD_595 nm_ of sample without TNF. The 20% inhibition concentration (IC_20_) was determined using a dose‐response nonlinear‐fit curve in GraphPad Prism v5.

### Surface plasmon resonance (SPR)

SPR experiments were performed with a Biacore T200 instrument using HBS‐P+ as the running and dilution buffer (GE Healthcare Bio‐Sciences). BIAevaluation software version 2.0 (GE Healthcare) was used for data processing. For determination of the binding kinetics, mouse anti‐human IgG (GE Healthcare) was covalently coupled to a CM5 Sensor Chip (GE Healthcare). Human antibody or DVD antibody was captured, and various concentrations of interaction partner protein were injected over the flow cell at a flow rate of 30 μl/minute. A double‐reference subtraction was performed using buffer alone. Kinetic rate constants were obtained by curve fitting according to a 1:1 Langmuir binding model.

### Immunohistochemical analysis

Formalin‐fixed paraffin‐embedded tissue sections were dewaxed, and antigens were retrieved after 10 minutes of boiling in citrate buffer, pH 6 (Dako). Slides were stained with 10 μg/ml of biotinylated DVD antibodies for 1 hour at room temperature and were visualized with streptavidin–HRP complex using 3,3′‐diaminobenzidine chromogen (Dako). Rabbit anti–ICAM‐1 IgG (Abcam) was detected with HRP‐conjugated goat anti‐rabbit IgG (Jackson Immunotools). Mouse anti–von Willebrand factor (Dako) and mouse anti‐CD31 (R&D Systems) were used to identify human vascular endothelial cells, which were revealed with HRP‐conjugated goat anti‐mouse IgG (Santa Cruz Biotechnology). Sections were counterstained with hematoxylin, mounted with Depex mounting medium (Dako), and acquired with a CellSens imaging system (Olympus).

## RESULTS

### Design and cleavage of an activatable dual variable‐domain antibody

**Figure 1:**
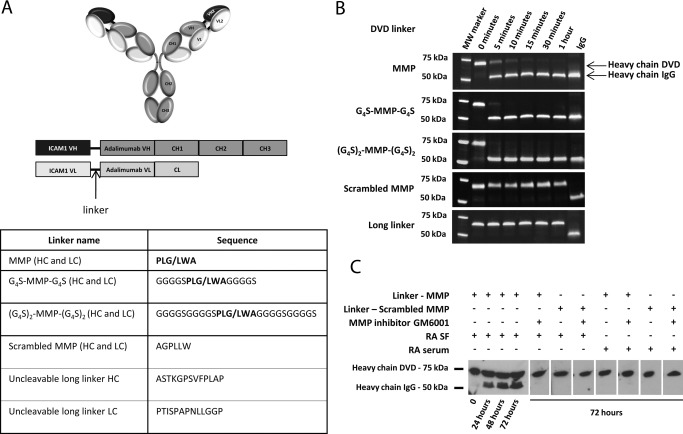
Structure and characterization of activatable dual variable‐domain (aDVD) antibodies. **A,** Schematic representation of the general structure of DVD constructs with an anti–intercellular adhesion molecule 1 (anti–ICAM‐1) outer domain linked to the anti–tumor necrosis factor (anti‐TNF) antibody adalimumab. Variable heavy (V_H_), constant heavy (C_H_), variable light (V_L_), and constant light (C_L_) chain regions are indicated. Linker length and amino acid composition are summarized in the table, with the matrix metalloproteinase (MMP)–cleavable sequence in boldface and the cutting position marked with a slash. HC = heavy chain; LC = light chain. **B,** Time course of aDVD antibody cleavage with recombinant MMP as resolved by sodium dodecyl sulfate–polyacrylamide gel electrophoresis. The gel shows a gradual conversion from DVD heavy chain to IgG heavy chain as a result of cleavage and removal of the outer anti–ICAM‐1 variable region. Similar processing was detected on the light chain (results not shown). **C,** Western blot analysis under reducing conditions. The time‐dependent increase in IgG heavy chain content due to cleavage of the biotinylated aDVD antibody carrying the short MMP‐cleavable linker, following incubation with rheumatoid arthritis (RA) synovial fluid (SF) at 37°C, can be seen. No cleavage was detected at 72 hours for the antibody carrying the scrambled MMP linker, nor was any cleavage detected upon incubation with RA serum or incubation in the presence of the MMP inhibitor GM6001.

To create a bispecific antibody format with therapeutic activity in RA and targeting capacity for the inflamed synovium, the gold standard for anti‐TNF biologic drugs, adalimumab, was coupled with an ICAM‐1–targeting antibody, using an adaptation of the well‐established DVD‐Ig format 17. The construct we describe contains the anti–ICAM‐1 V_L_ and V_H_ domains linked to the light chain and heavy chain, respectively, of the anti‐TNF drug adalimumab via a small peptide linker (Figure 1A). To create a DVD bispecific antibody with impaired binding capacity for the internal variable domain, a series of linkers with various lengths and amino acid compositions were designed to test for the desired activity (Figure 1A). The long linker was derived from a natural linker found in human IgG antibodies and was previously described in the context of the DVD‐Ig format 24. Reducing the linker length can substantially alter the kinetic properties of the internal binding domain 24.

We hypothesized that short linkers could impair the accessibility of the ligand to the internal domain in such a way that could be reverted upon cleavage of the internal linker, thus forming an activatable DVD prodrug. The remaining 4 linkers contained an MMP‐cleavable site (PLGLWA) 25, either alone or in the presence of G_4_S‐flanking regions, and a scrambled MMP‐cleavable sequence (AGPLLW). To test the ability of the MMP enzyme to access, cleave, and activate the internal anti‐TNF domain, the aDVD constructs where incubated with physiologically relevant concentrations of recombinant MMP enzyme. Analysis of the digested aDVD constructs by reduced SDS‐PAGE (Figure 1B) showed rapid processing of the aDVD carrying the MMP cleavable site, with the formation of molecular weight products consistent with an IgG format.

Incubation of the aDVD construct carrying the short MMP linker (PLGLWA) with SF from RA patients also showed time‐dependent activation of the construct, confirming the processing capacity under physiologic conditions (Figure 1C). Activation using SF was less efficient than that observed with recombinant protein. This may be due to saturation of MMP activity in ex vivo assays, which one would not anticipate to occur in vivo during chronic inflammation, where MMP expression in the synovial tissue is expected to be higher than in the surrounding SF 26. Additionally, the cleavage could be inhibited by the MMP inhibitor GM6001, while no cleavage could be detected for the aDVD carrying the scrambled MMP linker, further confirming MMP‐mediated activation of the constructs.

### Impaired binding of aDVD to TNF and rescue by MMP cleavage

**Figure 2:**
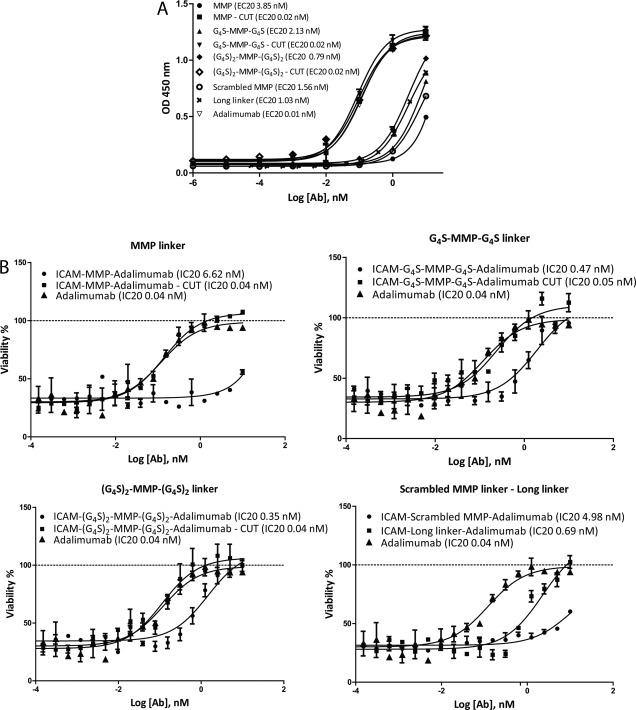
Anti‐TNF activity of DVD antibodies. **A,** TNF binding capacity for DVD antibodies (Ab) (scrambled MMP and long linker) and aDVD antibodies (MMP, G_4_S‐MMP‐G_4_S, and [G_4_S]_2_‐MMP‐[G_4_S]_2_), as determined by enzyme‐linked immunosorbent assay. Reduced binding capacity was detected for uncut aDVD constructs, while full potency could be restored upon cleavage with MMP enzyme, as compared with adalimumab IgG. **B,** Neutralization of TNF‐induced cytotoxicity in the L929 cell functional assay. Neutralization of cytotoxicity was impaired in uncut aDVD antibodies, with stronger impairment for ICAM‐MMP‐adalimumab antibody. Potencies similar to that of adalimumab IgG were obtained following MMP enzymatic digestion. Results in **A** are expressed as the 20% effective concentration (EC_20_) and results in **B** as the 20% inhibition concentration (IC_20_), corresponding to the dose necessary to obtain 20% of the activity. Values are the mean ± SEM. See Figure 1 for other definitions.

To be effective as a targeting prodrug, it is important that the aDVD molecules retain their ability to bind to their target antigen via the outer binding domain, while the inner domain remains shielded. The binding of aDVD molecules to ICAM‐1 (outer domain) and TNF (inner domain) was investigated via ELISA. The uncut aDVD molecules retained their binding to ICAM‐1 to the same extent as the parent anti–ICAM‐1 antibody (data not shown). However, before MMP cleavage, the molecules showed a 275‐fold reduction in binding to TNF as compared to adalimumab IgG. Binding to TNF was fully rescued for all the constructs following MMP cleavage (Figure 2A).

We used the L929 assay to assess the ability of the aDVD constructs to inhibit ligand binding to its receptor and to prevent TNF‐induced cytotoxicity. The ability of the uncleaved aDVD construct to block and inhibit TNF was severely impaired, consistent with the binding data obtained by ELISA. The uncleaved aDVD antibodies showed up to a 132‐fold increase in the IC_20_ as compared to adalimumab IgG, while cleavage with MMP completely rescued the inhibitory capacity (Figure 2B). As expected, the short MMP‐cleavable linker (PLGLWA) was characterized by a greater TNF binding impairment and was further validated using SPR.

**Figure 3:**
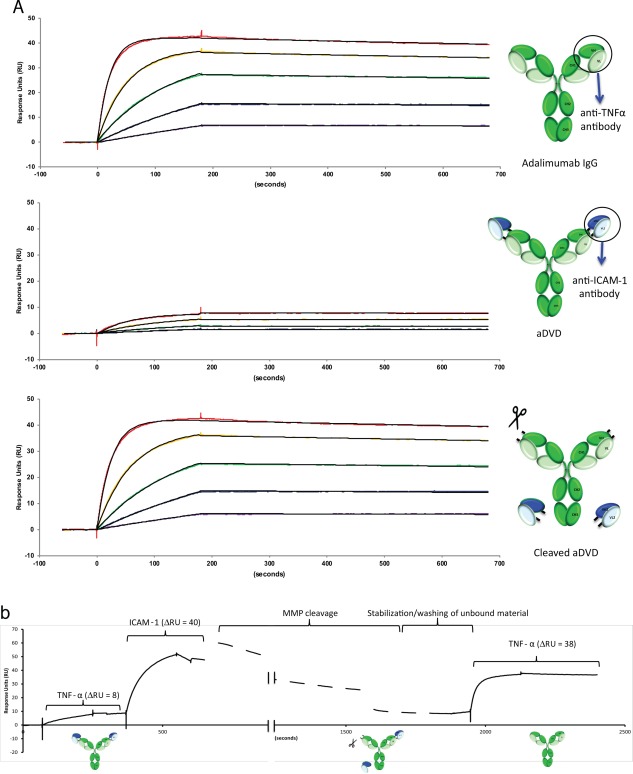
Analysis of the antigen‐binding kinetics of aDVD. **A,** Surface plasmon resonance sensorgrams showing the binding kinetics of adalimumab IgG, aDVD, and recombinant MMP–cleaved aDVD to TNF. The reduced binding capacity of aDVD for TNF could be reverted following digestion with MMP enzyme, restoring its full binding potential as compared to adalimumab IgG (see Table 1 for kinetic measurements). TNF concentrations were 20 n*M* (red), 8 n*M* (yellow), 3.2 n*M* (green), 1.28 n*M* (blue), and 0.512 n*M* (purple). **B,** Dynamic binding kinetics for TNF and ICAM‐1. When the aDVD antibody had been saturated with TNF of limited binding capacity, the second antigen was injected, showing retention of the ICAM‐1 specificity in the presence of TNF. On‐chip digestion of the construct with recombinant MMP enzyme was sufficient to cleave the antibody, releasing the outer domain and the coupled ICAM‐1 antigen. Injection of TNF at the end highlights the restored binding potency of the internal anti‐TNF domain. See Figure 1 for definitions.

We also compared the affinities of the restricted and processed forms of the aDVD molecule (Table 1). Binding of TNF to the uncleaved molecule was greatly impaired, as demonstrated by a 365‐fold reduction in the affinity constant (*K*_D_). Observation of the kinetics of binding indicated that the difference in affinity was predominantly driven by a reduction in the association rate constant (*K*_a_), which was 189‐fold lower than that for the uncleaved molecule, whereas the dissociation rate constant (*K*_d_) was largely unchanged (Figure 3A and Table 1). This result demonstrated that blocking of the external domain acts primarily by inhibiting association through steric hindrance; however, once bound, the antibody retains similar binding characteristics, indicating that the internal domain has not been modified and remains fully functional. Importantly, cleaved aDVD molecules showed not only identical affinity, but also identical component kinetics of binding to the parent adalimumab antibody (Table 1). In both cases, the binding kinetics were in good agreement with previously reported data 27.

**Table 1:** Surface plasmon resonance kinetic measurements of aDVD antibodies^a^

Molecule	Substrate	*K*_a_, 1/msec	*K*_d_, 1/second	*K*_D_, p*M*
Adalimumab IgG	TNF	2.416 × 10^6^	1.192 × 10^−4^	49.3
aDVD ICAM‐MMP‐adalimumab				
Cleaved	TNF	2.047 × 10^6^	1.087 × 10^−4^	53.1
Uncleaved	TNF	1.08 × 10^4^	1.83 × 10^−4^	18,000
Infliximab IgG	TNF	4.04 × 10^6^	2.5 × 10^−4^	61.8
aDVD ICAM‐MMP‐infliximab				
Cleaved	TNF	2.98 × 10^6^	3.66 × 10^−4^	123
Uncleaved	TNF	3.8 × 10^3^	6 × 10^−4^	158,000

a Activatable dual variable‐domain (aDVD) antibody constructs targeting intercellular adhesion molecule 1 (ICAM‐1) and the anti–tumor necrosis factor (anti‐TNF) antibodies adalimumab and infliximab were designed with matrix metalloproteinase (MMP)–cleavable linkers as described in Materials and Methods. *K*_a_ = association rate constant; *K*_d_ = dissociation rate constant; *K*_D_ = affinity constant.

In order to target proteins in a disease setting, the aDVD needs to maintain its cleavage capacity in the presence of both targeting and effector antigens, as is likely to be the scenario in the cytokine‐rich environment of the inflamed synovium. This is particularly pertinent as the aDVD is still capable of binding to TNF with a slow dissociation rate, which could conceivably block the cleavage site by steric hindrance (Figure 3A). In order to observe whether the aDVD molecule could be cleaved and activated in this environment, the molecule was immobilized on an SPR sensor chip, and saturating concentrations of TNF were injected, followed by ICAM‐1, prior to MMP cleavage on the sensor surface (Figure 3B). TNF showed the same restricted level of binding as had previously been demonstrated with the uncut material. ICAM‐1, however, was capable of binding to the molecule in the presence of TNF, as demonstrated by the observed change in response units, which was of the same magnitude as ICAM‐1 injected onto free antibody at the same concentration (data not shown).

In the presence of both saturating concentrations of ICAM‐1 and TNF, MMP enzyme was injected over a period of 30 minutes. Following chip cleavage and a period of stabilization to remove unbound material from the chip surface, the chip was rechallenged with TNF. Postcleavage, the TNF binding capacity was rescued, as demonstrated by the enhanced change in response units, which was measured at the same level as the injected concentration on the unrestricted antibody (Figures 3A and B).

### Tissue‐specific targeting using the aDVD antibody platform

**Figure 4:**
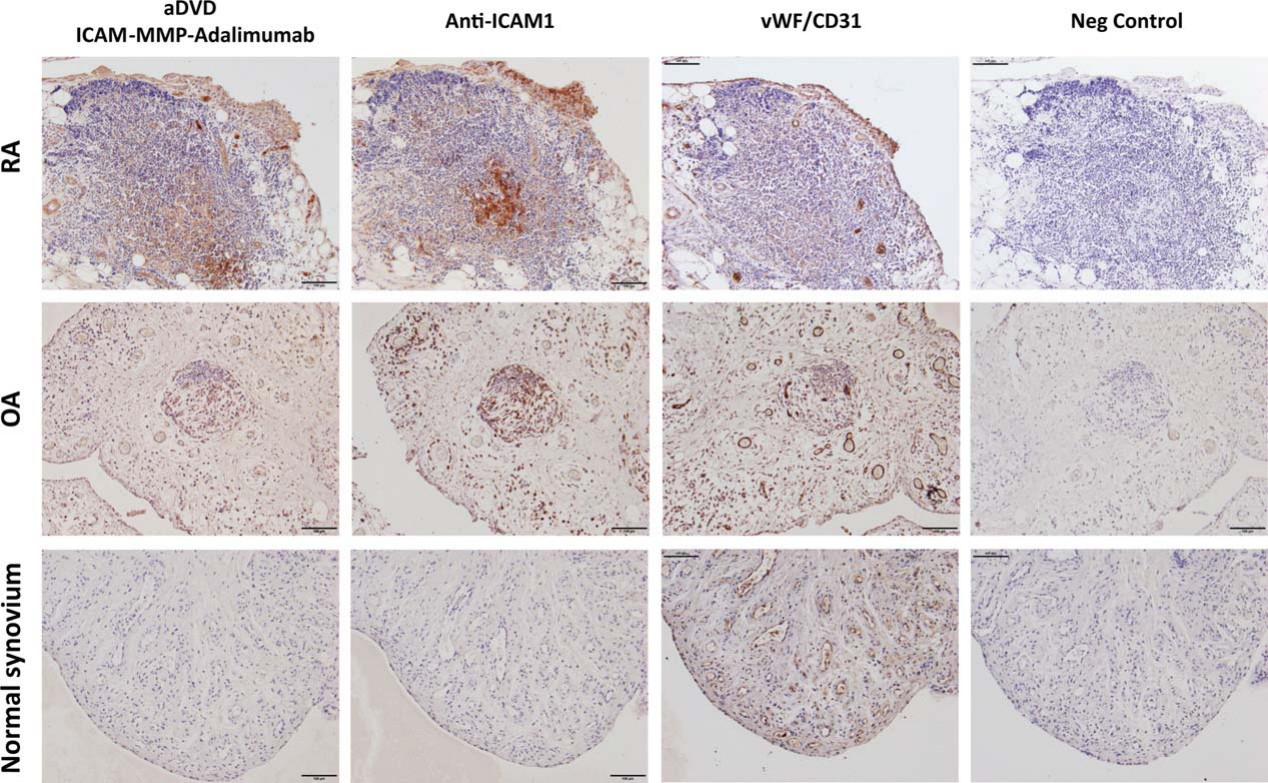
Human synovial tissue reactivity of aDVD antibodies. The reactivity of the ICAM‐1‐MMP‐adalimumab construct with RA, osteoarthritis (OA), and nonarthritic (normal) human synovial tissues was examined using immunohistochemistry. Bound biotinylated aDVD antibodies were detected using streptavidin–horseradish peroxidase complex and compared to the staining pattern of anti–ICAM‐1 IgG. The presence of blood vessels was depicted using anti–von Willebrand factor (anti‐vWF) antibody in combination with anti‐CD31 antibody. Biotinylated human IgG was used as the negative control. Bars = 100 μm. See Figure 1 for other definitions.

One of the key characteristics of the aDVD format is the ability to present the anti‐TNF therapeutic function in a prodrug format that can be activated following encounter with MMP enzymes at the site of arthritic inflammation. The presence of the outer variable‐domain–targeting ICAM‐1, an integrin overexpressed in inflammatory conditions such as RA 28, 29, would allow the preferential accumulation of antibody in the target tissue, facilitating the encounter with proteolytic enzymes. MMP cleavage causes the removal of the anti–ICAM‐1 external domain, resulting in loss of ICAM‐1 specificity (data not shown). To test the ability of the aDVD to retain tissue‐targeting capacity when in full conformation, we examined its reactivity with the microvasculature in samples of human synovium obtained from 3 RA patients, 3 osteoarthritis patients, and a patient without arthritis (normal synovium) via immunohistochemistry (Figure 4).

The ICAM‐MMP‐adalimumab aDVD was able to selectively target the human inflamed synovium from both the RA patients and the OA patients with similar efficacy as compared to an anti–ICAM‐1 IgG antibody. Importantly, no detectable reactivity was identified in the normal synovium sample from a patient without arthritis. The specificity for arthritic synovium further strengthens the potential of the aDVD for use in targeted drug delivery in RA.

Furthermore, the aDVD format may represent a flexible platform for targeted delivery of prodrugs that can be easily adapted to other cytokines and to other disease conditions with a simple exchange of the outer targeting domain.

### Improvement in the structural design of aDVD molecules with knowledge of the molecular interactions

**Figure 5:**
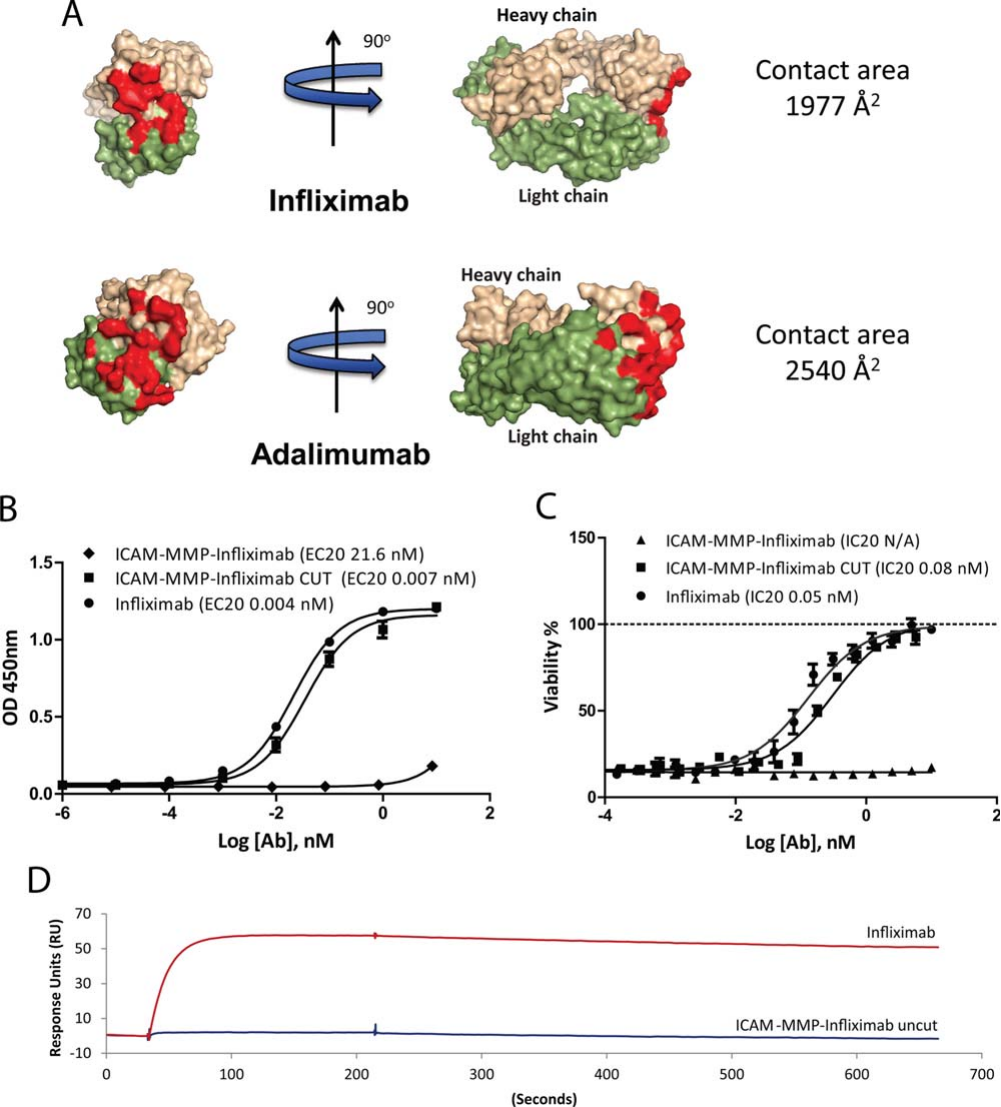
Molecular interactions as determinants of aDVD antibody (Ab) inhibitory properties. **A,** Schematic representation of the interaction of infliximab and adalimumab with TNF. There is a reduced contact area (red) for infliximab as compared to that for adalimumab, which may predict an increased binding inhibition in the aDVD format. Adapted, with permission, from ref. 31 (© 2013 The American Society for Biochemistry and Molecular Biology). **B,** TNF binding capacity for the aDVD ICAM‐MMP‐infliximab construct. Binding capacities before and after MMP cleavage were compared to that of infliximab, as determined by enzyme‐linked immunosorbent assay. A 2,500‐fold inhibition for the infliximab aDVD construct compared to infliximab was observed, with rescue of the binding upon cleavage of the construct with MMP. **C,** Neutralization of TNF‐induced cytotoxicity in the L929 cell functional assay. There was a complete loss of function of the ICAM‐MMP‐infliximab construct as compared to infliximab. Results in **B** are expressed as the 20% effective concentration (EC_20_) and results in **C** as the 20% inhibition concentration (IC_20_). Values are the mean ± SEM. **D,** Binding kinetics of aDVD ICAM‐MMP‐infliximab and infliximab to TNF. Results show the binding of 20 n*M* TNF to infliximab and ICAM‐MMP‐infliximab coupled to the sensor surface at the same density. See Figure 1 for other definitions.

Since the reduced binding of aDVD molecules can be mediated by blocking of the internal domain, we hypothesized that further inhibition of binding could be predicted through knowledge of the interaction between TNF and the inner domain antibody. The crystal structures of adalimumab and infliximab in complex with TNF have recently been reported 30, 31. Crystal structure data showed that adalimumab bound to trimeric TNF via a broader binding interface, with a total buried surface area of 2,540 Å^2^ 31, while infliximab bound to the TNF trimer via a reduced binding interface of 1,977 Å^2^ (Figure 5A). Additionally, adalimumab engages the TNF trimer through interactions with 2 monomers of the trimer, while the binding of infliximab is mediated almost exclusively through the loop region of a single TNF monomer. We therefore predicted that the smaller interaction surface area in the infliximab–TNF complex would translate to a binding interface that would be more readily blocked by the outer domain. To test this, an aDVD molecule was engineered with infliximab as the inner binding domain and was tested for binding and functionality.

Infliximab bound to TNF with an EC_20_ of 0.004 n*M*, while the aDVD‐infliximab bound with an EC_20_ of 21.6 n*M*. Once processed by MMP cleavage the antibody demonstrated binding that was comparable to the original infliximab antibody (Figure 5B). The fold difference between cleaved and uncleaved aDVD‐infliximab antibody was 3,000, which was 10‐fold higher than the difference measured for the aDVD‐adalimumab construct. The ability of the antibody to inhibit TNF binding to its receptor in L929 cell functional studies was also greatly diminished, as no anti‐TNF functionality could be detected for the uncleaved aDVD‐infliximab over the range of concentrations tested, while activity was fully rescued after linker cleavage (Figure 5C). SPR data demonstrated a 2,500‐fold reduction in the *K*_D_ that was predominantly driven by a reduction in the *K*_a_, with an observed 1,000‐fold reduction in the association constant (Figure 5D and Table 1). These data further demonstrated the flexibility with which different binding moieties can be introduced and highlighted the fact that the molecular interactions between parent antibody and the target antigen can be used to design aDVD molecules with more‐potent blocking capacity.

## DISCUSSION

The last 2 decades have marked a substantial revolution in the treatment paradigm for RA. The advent of biologic agents has provided a new avenue for the successful treatment of RA. However, there remain a considerable number of patients who do not respond to the available therapies and in whom a treatment‐free remission is rarely achieved 5, 6. RA also represents one of the most lucrative markets for pharmaceutical companies, and in 2013, the 3 top‐selling drugs were all biologic agents marketed for the treatment of RA (see EvaluatePharma, which is available online at http://www.evaluategroup.com/). Despite the obvious success of the current treatments, little effort has been invested in improving the safety profiles of the available therapeutic alternatives.

The identification of tissue‐specific or overexpressed antigens in the inflamed synovium may provide a solution to these concerns and allow the development of therapeutic agents for tissue‐specific drug delivery 32. Several candidates are now being considered for arthritic synovium targeting, including the oncofetal extradomain A of fibronectin. Indeed, a single‐chain antibody fragment that targets extradomain A has recently entered clinical evaluation as an scFv‐IL‐10 fusion protein for the treatment of RA (ClinicalTrials.gov ID NCT02076659). An alternative strategy could involve the use of bispecific antibody to combine tissue targeting with therapeutic function. To date, no bispecific antibody has been clinically evaluated for use in RA; however, 2 antibodies, catumaxomab and blinatumomab, have been recently approved for cancer treatment, and several constructs are currently in clinical trials 33.

The bispecific antibody format DVD‐Ig has shown potential as a versatile platform for dual‐antigen targeting 17. One of the crucial aspects necessary for a conventional bispecific antibody is the capacity for real synergistic activity between the 2 binding moieties. Differences in binding affinities may result in targeting skewed toward one antigen and, as a consequence, suboptimal therapeutic activity. This is probably the main drawback when combining tissue‐targeting moieties with existing therapeutic domains, which are usually characterized by very high affinities. Interestingly, the size and composition of the linker between the outer and inner variable domain of DVD‐Ig antibodies has been shown to significantly affect the kinetic activity of the inner region 19. We hypothesized that by reducing the linker length, we could selectively impair antigen accessibility to the internal domain in a reversible manner, via the presence of an MMP‐cleavable site within the linker. By placing the therapeutic moiety on the inner region, we could obtain 3 important effects: 1) binding capacity skewed toward tissue targeting provided by the outer variable domain, 2) inhibition of systemic engagement of the inner therapeutic binding region, and 3) selective activation of the therapeutic antibody at the site of local inflammation. This construct would therefore provide a tissue‐specific delivery of an antibody prodrug.

We developed an activatable DVD‐like construct with an anti–ICAM‐1 outer domain (for targeting the inflamed synovium) linked to the anti‐TNF antibody adalimumab. The linkers we designed contained an MMP‐cleavable sequence and were readily cleaved by the proteolytic MMP‐1 enzyme, both in recombinant form and in physiologic form in human RA SF, providing insight for efficient in vivo antibody activation. Although MMP overexpression is generally associated with inflammation, angiogenesis, and wound repair, different tissues/conditions are characterized by increased expression of specific MMP subgroups. In the context of inflammation, elevated levels of MMPs 2, 7, 8, and 9 have been reported in experimental autoimmune encephalomyelitis 34, 35, 36, while MMPs 3 and 9 have been associated with cutaneous inflammation 37. In inflammatory arthritis, overexpression of MMPs 1, 3, 9, and 13 has been correlated with disease progression and joint damage 26, 38, 39. MMP levels in RA SF and serum were found to be significantly higher than those in healthy controls, with SF levels being several hundred‐fold higher than serum levels 39, 40.

As synoviocytes in the lining layer represent the predominant source of MMPs in the arthritic synovium 41, the synovial tissue can be expected to display a greater concentration of MMP‐1 and MMP‐3 than the associated SF and peripheral blood. The slower antibody cleavage rate observed when using RA SF and the lack of activation in RA sera may result in an advantage in vivo, where only the antibody that is actively accumulated in the arthritic synovial tissue may be held long enough for efficient MMP cleavage. Furthermore, in the context of infections such as tuberculosis (a major risk of treatment with anti‐TNF), the main driver of cartilage degradation is MMP‐9, with low secreted levels of MMP‐1 (100‐fold lower than in RA SF) 42, 43. This may result in increased safety because of a reduced risk of unwanted antibody activation in other tissues, and/or it may result in the presence of concomitant infections.

Characterization of the binding of TNF by ELISA and inhibition of the biologic activity of TNF in L929 cell assays proved that short linkers could substantially restrict antigen accessibility and binding capacity. The binding, however, could be fully resolved upon MMP cleavage. Among the 3 MMP‐containing linkers, the short PLGLWA linker showed the highest binding inhibition, reasonably due to the vicinity of the outer domain and increased steric hindrance. Indeed, measurement of the binding kinetics by SPR showed impaired antigen access to the inner domain, resulting in a slower *K*_a_. Once bound, however, the *K*_d_ kinetics for TNF were unchanged, suggesting unaltered functionalities of adalimumab in the aDVD format. This was further confirmed by the identical kinetics of the cleaved aDVD and the parent adalimumab antibody. This effect was more pronounced when we used variable regions with smaller binding interface (e.g., infliximab).

The change of paradigm in the treatment of RA with the consequent huge costs to national health systems has spurred the identification of new clinical tools for stratification of patients in order to improve therapeutic effectiveness and reduce adverse effects as well as costs 44. The next step in increasing the therapeutic potency and the safety profile would be the development of tissue‐specific (not only inflammation‐specific) targeting agents 32. With regard to RA synovium, our group of investigators has previously identified peptides and scFv antibody fragments with selective specificity for the synovium 45, 46, 47. The incorporation of the scFv A7 in the aDVD format, as demonstrated herein for ICAM‐1, will provide specificity for human arthritic synovium to the molecule, with great potential for tissue‐specific drug delivery in RA.

These constructs have the potential to increase safety because of their selective activation in situ and their inhibited systemic cytokine engagement and to increase potency because of their enhanced therapeutically relevant concentrations achieved at the site of active disease. We can believe that the development of tissue‐targeting prodrugs such as the aDVD described herein could represent optimum flexible platforms for the rational design of therapeutic agents with significant impact on RA.

## AUTHOR CONTRIBUTIONS

All authors were involved in drafting the article or revising it critically for important intellectual content, and all authors approved the final version to be published. Dr. Pitzalis had full access to all of the data in the study and takes responsibility for the integrity of the data and the accuracy of the data analysis.

**Study conception and design.** Onuoha, Ferrari, Sblattero, Pitzalis.

**Acquisition of data.** Onuoha, Ferrari.

**Analysis and interpretation of data.** Onuoha, Ferrari, Sblattero, Pitzalis.
